# Developmental characteristics of Williams-Beuren syndrome and evaluation of adaptive behavioral skills

**DOI:** 10.55730/1300-0144.5701

**Published:** 2023-09-09

**Authors:** Şenay GÜVEN BAYSAL, Feyzullah Necati ARSLAN, Mehmet Akif BÜYÜKAVCI, Fatma Hilal YAĞIN, Cemal EKİCİ, Zeynep ESENER, Derya GÜMÜŞ DOĞAN

**Affiliations:** 1 Department of Developmental Pediatrics, Gazi Yaşargil Training and Research Hospital, Diyarbakır, Turkiye; 2 Department of Developmental Pediatrics, Necip Fazıl City Hospital, Kahramanmaraş, Turkiye; 3 Department of Developmental Pediatrics, Faculty of Medicine, İnönü University, Malatya, Turkiye; 4 Department of Biostatistics and Medical Informatics, Faculty of Medicine, İnönü University, Malatya, Turkiye; 5 Department of Medical Biology and Genetics, Faculty of Medicine, İnönü University, Malatya, Turkiye; 6 Department of Medical Genetics, Gazi Yaşargil Training and Research Hospital, Diyarbakır, Turkiye

**Keywords:** Williams-Beuren syndrome, developmental behavioral pediatrics, adaptive behavior, maladaptive behavior, developmental monitoring

## Abstract

**Background/aim:**

Williams-Beuren syndrome (WBS) is a rare genetic disorder with delays in language and cognitive development, but, with increased awareness of clinical features and a reliable diagnostic test, WBS is becoming more widely recognized in childhood. Adaptive behavior skills and/or maladaptive behavior are important for the prognosis of individuals with WBS. The aim of this study was to investigate the clinical and developmental characteristics of patients with WBS and further increase awareness about it by evaluating the adaptive skills and maladaptive behaviors of the patients.

**Materials and methods:**

The data of WBS patients followed-up at the Developmental Behavioral Pediatrics Unit were reviewed. Patient data on perinatal and postnatal history, developmental stages, physical and neurological examination findings were collected. The International Guide for Monitoring Child Development (GMCD) was administered to each child. In addition, semistructured interviews were conducted with the parents using the Vineland Adaptive Behavior Scales, Second edition (Vineland-II).

**Results:**

A total of 12 patients diagnosed with WBS via detection of the 7q11.23 deletion, of whom 6 were girls, were retrospectively reviewed. The mean age at the time of review was 54.6 ± 32.5 months. The mean age at first presentation to the Developmental Behavioral Pediatrics Outpatient Clinic was 15 ± 11.5 months. In the first developmental evaluation using the GMCD, there was a delay in fine and gross motor domains in 6 patients, in the language domains in 4 patients, and in all of the domains in 2 patients. Findings with Vineland-II showed socialization and communication domains as strengths, but the daily living skills and motor skills domains were weaknesses. In terms of maladaptive behavior, the patients tended to frequently have behavioral problems, neurodevelopmental disease, anxiety disorders, eating problems, and sleeping problems.

**Conclusion:**

This retrospective review of 12 patients indicated a general delay in overall development, and confirmed impairment in both adaptive and maladaptive functioning in WBS.

## 1. Introduction

Williams-Beuren syndrome (WBS) is a genetic disorder with delays in language and cognitive development, with a reported incidence of around 1 in 7500 people. WBS constitutes approximately 6% of genetic causes of intellectual disability and features may include dysmorphic facial appearance, developmental delay, hypotonia, congenital heart disease, hypercalcemia, chronic otitis media, strabismus, hyperacusis, inguinal hernia, gastroesophageal reflux, constipation, short stature, endocrinological disorders, and connective tissue anomalies. WBS is characterized by typical personality and cognitive problems, including high empathy, extreme warmth, attention problems, and anxiety [1–3]. In most cases, the physical symptoms are subtle and go unnoticed in infants and young children, so diagnosis may be delayed [7]. Children with WBS also have behavioral problems that include difficulties eating and sleeping [8, 9].

WBS is known to be caused by a microdeletion affecting chromosome 7, including the elastic gene, and 99% of patients have a submicroscopic deletion of 7q11.23 detected on fluorescent in situ hybridization (FISH). WBS was initially thought to be a rare genetic disorder but has emerged as one of the more widely recognized genetic disorders in childhood, with increasing awareness of clinical features and the emergence of a reliable diagnostic test [10]. It has been proposed that WBS is one of the diseases that links genes, the brain, cognitive functions, and behavior [11]. WBS is characterized by developmental and language delay, intellectual disability, difficulty with conceptual vocabulary, executive control deficits, and impairment in language that vary according to the level of intellectual functioning [12]. However, in WBS the cognitive profile is heterogeneous, so that the degree of deficits or relatively well-preserved abilities will result in contrasting neurocognitive clinical assessment findings between individual patients [13]. Adaptation functions include communication, self-care, home life, interpersonal skills, use of community opportunities, self-direction, functional skills related to school, work and leisure time, and health and safety.

In this study, the aim was to report the clinical and developmental characteristics of patients with WBS followed-up at a single Turkish tertiary level center and evaluate the range of exhibited adaptation behavior in order to increase awareness of the disease. This study used Vineland-II to investigate the adaptive and maladaptive behaviors of children with WBS. It is hoped that earlier diagnosis will enable more rapid intervention and treatment of developmental and behavioral problems.

## 2. Materials and methods

All patients who were followed-up with the genetically confirmed diagnosis of WBS in the Department of Developmental Pediatrics of İnönü University Faculty of Medicine were retrospectively reviewed. In the study, the amount of Type I error (alpha) was 0.05, the power of the test (1-beta) was 0.8, and the effect size was 0.67, while the minimum sample size required according to the theoretical power analysis process applied using the correlation analysis should be 12 patients^1^. Ethics committee approval was obtained from İnönü University Health Sciences Research and Publication Ethics Committee (Approval no.: 10/01/2022-E. 130696). The inclusion criteria were being patients who were followed-up in the developmental pediatrics outpatient clinic with a diagnosis of WBS and having families who agreed too voluntarily participate in the study. Patients who were previously in our follow-up and did not continue with it were excluded from the study. Participation in the study was approved by the primary caregiver after giving written informed consent. Patient data were collected from past medical records. For the purposes of this study, the age at diagnosis was considered to be the time when the genetic diagnosis was made. Data regarding perinatal and postnatal history, developmental stages, and physical and neurological examination findings were obtained from patients’ medical records.

The procedure for genetic diagnosis was as follows. Peripheral blood (2 cc) was collected from the patients and 24-h cell culture was performed using peripheral blood cell culture medium (BIO-PBTM Karyotyping Medium: Kibbutz Beit Haemek, 25115, Israel). After harvesting, a WBS microdeletion FISH probe (Cytocell Williams-Beuren, Milton, Cambridge, UK) was hybridized, 200 interphase cells were analyzed using a fluorescent microscope, and patients with microdeletion syndrome were identified.

A medical history form was filled in by the family of each child during their first visit to the Developmental Pediatrics Unit. The informants were responsible for the child’s care and were all primary caregivers. The interviews ranged in length from 45 min to 3 h, with a mean duration of 1.5 h. The form included basic sociodemographic information, current and/or past health problems, and questions about WBS. The International Guide for Monitoring Child Development (GMCD) was applied to each child. The GMCD was developed by Ertem et al. in Turkey [14]. The GMCD provides developmental monitoring and early detection of developmental difficulties in low- and middle-income countries. The GMCD consists of a brief, open-ended, precoded interview with the caregiver of children younger than 42 months. The first question is about parental concerns. Subsequently, open-ended questions are asked about the developmental areas of expressive language, receptive language, gross and fine motor skills, relating to others, play activities, self-help activities (for children over 12 months). The caregiver’s responses to the questions are used to identify appropriate developmental milestones for each child with delay in developmental domains being defined according to the GMCD. Milestones were placed in the appropriate age columns in the international standardization protocol. Interpretation of the results is as follows. All milestones in the age column are attained by ≥85% and not attained by <15% (approximating < −1 standard deviation (SD)) of healthy children. All milestones before the age column are attained by ≥95%–97% and not attained by < 5%–3% (approximating < −2 SD) of healthy children. This assessment has a sensitivity of 0.71–0.94 and specificity 0.69–0.82 [15]. The GMCD is the most widely used scoring tool among 27 developmental assessment tools used globally in children <2 years and covering ≥3 domains [16].

The use of adaptive behavior skills is critical for the prognosis of WBS. These types of behaviors include the skills individuals need to function and be self-sufficient in their everyday environment [17]. The Vineland Adaptive Behavior Scales, Second Edition (Vineland-II) provides very useful and detailed information for clinicians when the adaptive functionality of individuals in all age groups is determined. There are 4 domains and 11 subdomains evaluated by Vineland-II, comprising communication (expressive language, receptive language, and written language); daily living skills (personal, domestic and community); socialization (interpersonal relationships, play and leisure, coping skills); and motor skills (gross motor and fine motor). In addition, there are 3 subdomains covering maladaptive behaviors, including internalization, externalization, and other. The original Vineland-II was revised by Sparrow et al. in 2005 [17], and translated and validated for Turkish children by Alpas et al. [18]. In the study of Akçakın et al. [19], Vineland-II was given to a total of 553 children, comprising 274 females and 279 males. The results showed that as the children got older, their total scores from the scale also increased. The reliability of the scale was determined by internal consistency analysis and was found to be 0.68–0.97. The results showed that Vineland-II can be used as a quantitative and qualitative assessment tool for Turkish children in infancy, early childhood, and school age period^2^. They suggested that there was a strong theoretical and empirical connection between the content of the test and behavior or use of skills. A similar finding was shown with the original form of the scale.

Researchers have GMCD and Vineland-II practitioner training and these tools are regularly used in developmental pediatric outpatient follow-ups. Vineland-II raw scores can be converted to Vineland-II standard, V-scale scores. Standard scores range from 20 to 160 (with a population mean of 100 and a SD of 15). The subdomains have scaled scores called V-scaled scores, which range from 1 to 24 (with a population mean of 15 and a SD of 3). Results can be categorized by adaptive levels and maladaptive levels. Adaptive levels are described as high (standard score range: 130 and above), moderately high (115–129), adequate (86–114), moderately low (71–85), and low (70 and below). Maladaptive levels are also subcategorized into average (V-scale score range below 18), elevated (18–20), or clinically significant (21–24) [17].

### 2.1. Statistical analysis

Qualitative variables were expressed as frequency and percentages. The conformity of the quantitative variables to normal distribution was examined using the Shapiro-Wilk test. Quantitative variables satisfying the assumption of normal distribution were expressed as the mean and SD, and nonparametric quantitative variables were expressed as the median (range). The Mann Whitney-U test was used in comparison of the quantitative data according to GMCD status since parametric test assumptions were not provided. The Pearson correlation coefficient was calculated to examine the relationships between the scale scores. P < 0.05 was considered significant. All of the statistical analyses were performed using IBM SPSS Statistics for Windows 26.0 (IBM Inc., Armonk, NY, USA).

## 3. Results

Retrospectively reviewed were 12 children, comprising 6 females and 6 males, who had been diagnosed with WBS by detecting the 7q11.23 deletion at the Department of Medical Genetics. The mean age of the children upon inclusion into the study was 54.6 **±** 32.5 months, while the mean age at diagnosis was 12.8 **±** 7.8 months, ranging from 1 to 27 months. Of the patients, 2 had a history of prematurity. Only 3 had a history of normal delivery. The mean birth weight of all of the patients was 2512.5 ± 366.8 g. The average time to the first presentation at developmental pediatrics was 15 **±** 11.5 months. Departments referring the patients were general pediatrics (n = 4, 33.3%), genetics (n = 4, 33.3%), pediatric neurology (n = 2, 16.7%), and pediatric cardiology (n = 2, 16.7%). Moreover, 4 patients had attended developmental pediatrics prior to genetic diagnosis, all of whom had been referred to the Genetics Outpatient Clinic by the Developmental Behavioral Pediatric Unit with a preliminary diagnosis of WBS.

All 12 patients had facial dysmorphism. Dysmorphism was characterized by a wide forehead, periorbital fullness, a wide mouth, a rounded nose, flattening of the nasal root, full cheeks, and small teeth with evident gaps. Patient medical histories included failure to thrive, height and weight <5th percentile, hypersensitivity to sound, visuospatial problems, ocular and visual findings, chronic otitis media, hearing loss, and delayed speech acquisition followed by excessive talking. Furthermore, systemic problems were also evident, including cardiovascular, gastroesophageal, endocrinological, genitourinary, connective tissue, and musculoskeletal system problems. The clinical features of all of the WBS patients are shown in Table 1.

The children started special education programs at an average age of 21.05 ± 12.9 months, ranging from 2 to 47 months. One received only individual training, 1 received only physical therapy rehabilitation, and 7 had both individual and physical therapy rehabilitation. Progress in all areas of development was reported in the children included in the special education program in the early period. In 2 patients, special education was started too late due to late acceptance by the family. The patient with autism-like symptoms and his family were provided intensive communication-based developmental support and started a special education program quickly. After starting the special education program, there was a significant improvement in the field of communication and relationship with this child. Additionally, 2 children attended kindergarten and 3 attended special education classes. When the families whose children did not go to special education were asked about their reasons, all of them cited the COVID-19 pandemic.

The children attended 6 (range 2–13) developmental pediatric outpatient follow-ups. with the first developmental evaluation occurring at a median age of 12.6 (2–19) months. The results of the first GMCD showed delay in gross and fine motor skills (n = 6), delay in the language area (n = 4) and the remaining 2 children had delay in all areas. Thus, 75% of the children had developmental delays in a developmental domain or domains evaluated by the GMCD.

In addition, these children were evaluated via a semistructured interview with the parents using Vineland-II. The scores for Vineland-II are shown in Table 2. Based on the characteristic cognitive and personality profiles of children with WBS, it was predicted that socialization and communication areas would be strengths, while daily living skills and motor skills would be weaknesses. In addition, there was a negative correlation between the age at diagnosis and communication standard score, daily living skills standard score, socialization standard score, and total adaptive behavior composite standard score, wherein the scores decreased as the age at diagnosis increased. The Figure shows a significant relationship between the variables communication V-scale score standard score, daily living skills standard score, socialization standard score, and motor skills V-scale score (p ≤ 0.005).

When the families were interviewed about the maladaptive behaviors of their children, there were frequent reports of behavioral problems, neurodevelopmental disease, anxiety disorders, eating problems and sleep problems. Moreover, 4 patients were followed-up with a diagnosis or diagnoses of autism spectrum disorder, attention deficit hyperactivity disorder and obsessive-compulsive disorder. These are shown in Table 3. There was a significant increase in maladaptive behaviors in those children with concomitant neurodevelopmental diseases, as given in Table 4. In addition, the Vineland-II adaptive scores were lower in patients who were delayed in any area in their developmental evaluation with the GMCD, as seen in Table 5.

## 4. Discussion

Early diagnosis of WBS allows for earlier detection and treatment of developmental, behavioral, and medical problems. Huang et al. [7] reported the mean age at diagnosis of WBS to be 3.66 years (SD 4.13), while the mean age at first concern was 0.98 years (SD 1.24), resulting in a diagnosis delay of 2.77 years (SD 4.10). They observed a lengthy delay in the diagnosis of WBS but noted that the involvement of a geneticist was associated with earlier diagnosis and a reduced number of tests. In the present study, the mean age at diagnosis was 12.8 **±** 7.8 months. This earlier diagnosis may have been due to follow-up of the patients at a university hospital, coupled with a detailed evaluation of patients with dysmorphic features in the developmental pediatrics department and the rapid genetic counseling. Delay in the diagnosis of WBS has important clinical implications, as later diagnosis may affect morbidity and prognosis [20]. In addition, failure to recognize individuals with WBS can negatively impact a child’s education and long-term outcomes [21]. In the cohort described in the present study, the GMCD and Vineland-II were used, since the study aimed to monitor indicators of developmental delay and adaptive-maladaptive behaviors.

Braga et al. evaluated 8 children with WBS, aged 48 to 72 months, using the Denver test, and developmental delay was found in 100% of them. Although developmental delay was detected for all 4 Denver subscales, the children achieved better results in the language and gross motor scales [5]. Kirchner et al. evaluated the development of 16 WBS children, aged 3 months to 5 years, with the Bayley Scales of Infant and Toddler Development, Third Edition (Bayley-3). In their study, the Bayley-3 scores for motor skills were significantly lower than those of both the cognitive and language skills. The results showed delays in all domains, where children with WBS scored more than 1 SD below the mean in each domain [22]. Similarly, in the present study, delays were especially detected in the evaluation of motor skills with the GMCD.

It has been reported that 75% of children with WBS have intellectual disability [4, 5]. The WBS cognitive profile progresses with language development, intellectual disability including extreme weakness in short-term memory and visual-spatial perception, and difficulties in interpersonal relationships and daily living skills. Various studies have been conducted into the cognitive weaknesses and strengths of children with WBS [2–4]. It has been reported that auditory short-term memory is more advanced than the general intellectual ability. Language skills are in line with or slightly better than general intellectual ability. However, there is extreme weakness in visual-spatial abilities, such as block design, pattern making, or drawing tasks, and this has been confirmed by several studies. This weakness was also reported by the families of 9 of the children in the present study with problems with painting skills, drawing skills, puzzle completion, 3-dimensional thinking, and imaginative visualization skills being reported.

A characteristic behavioral personality profile has also been described for people with WBS. Patients are consistently described as being overly friendly. This overly friendly trait includes behaviors such as being more likely to initiate interactions with other people, over-enthusiasm to approach and interact with strangers, and a lack of shyness towards strangers [23]. These traits were evident in 10 of the 12 children in the present study too, based on clinical observations and family statements.

A study by Gosch and Pankau discussed the adaptive behavior of children with WBS. They compared the adaptive behavior of children with WBS aged 4 to 11 years (n = 19) with that of children with nonspecific intellectual disabilities (n = 19). The children were matched for sex, cognitive ability, and chronological age. The results showed that the children in the WBS group had lower adaptive behavior ability. The difference between the groups was due to the greater weakness in both fine and gross motor skills in the WBS group [24]. Greer et al. studied the adaptive behaviors of 15 children with WBS, aged 4 to 18 years, using the Vineland Adaptive Behavior Composite and it was reported that the children’s communication and socialization skills were significantly better developed than their daily living skills [21]. These 2 studies show that children with WBS are relatively weak in daily living skills. In line with this, patients in the present study were reported by their families to have weaknesses, especially in fine motor skills, that negatively affected daily life skills.

The Vineland Adaptive Behavior Scales-Interview Edition was used in a study by Mervis et al. with 41 children with WBS, aged 4–8 years [25]. Once again, it was found that socialization and communication areas were relatively strong, while daily life skills and motor skills were relatively weak. Socialization skills were reported to be stronger than communication skills, and interpersonal skills were better than play/leisure or coping skills in the socialization area. Skills in the motor area were found to be the weakest. Performance in daily living skills was found to be quite weak compared to performance in other areas. The Adaptive Behavior Standard score was not found to be associated with chronological age. Hahn et al. evaluated the adaptive behavior profile of 18 children with WBS under 5 years of age (mean age of 47.6 months) using the Vineland Adaptive Behavior Scale. Significantly higher scores were reported in the communication domain compared to the daily living domain, the socialization domain had a significantly higher average score than the daily living domain and the motor domains, while the communication domain had a significantly higher average score than the motor domain [26]. Braga et al. evaluated 8 children of both sexes with WBS, aged 48 to 72 months [5]. In WBS, major developmental disorders have been associated with poorer fine motor skills and self-care skills. The main consequences on the Vineland scale are losses in areas of socialization (interpersonal relationships, social skills, play and leisure activities) and daily living activities. The biggest difference between adaptive performance age and chronological age was shown in the adaptive functionality in daily life domains (personal, domestic, and social), followed by adaptive functionality in socialization domains (interpersonal relations, play and leisure, social skills). In the present study, it was found that skills were delayed in all areas correlated with age, and coping skills were poorer in daily life skills and socialization. When the skills in the field of motor development were assessed, it was seen that the limitation in fine motor skills was the most striking.

The strengths of this study were that WBS is in the rare disease group and the follow-up of these patients was performed with a transdisciplinary approach at our Developmental Pediatrics Unit. All of the patients were evaluated with a structured assessment that included a family-centered and holistic approach. This was an indispensable opportunity for the children and their families. Standardized tools such as the Vineland-II and the GMCD, which have accepted validity and reliability in the world literature, were used in the evaluation. The main limitation of this study was the small sample size and the fact that it was conducted at a single center in eastern Türkiye, so the data obtained could not be generalized to all of Turkey. Our future goal is to increase the sample size and obtain more generalizable results with multicenter studies to be conducted in Türkiye.

In summary, for the follow-up of children with WBS, it is recommended to take a detailed disease history, predict possible clinical complications, and perform age-appropriate periodic follow-ups [5]. Since cardiac, urologic, neurologic, and endocrinologic problems that frequently accompany WBS in patients are of vital importance, the follow-up and evaluation of cognitive development may be ignored in these patients while dealing primarily with these problems. Although this study was conducted with a small sample, there was evidence of impaired cognitive and adaptive functioning and an increase in maladaptive behaviors within the group. We believe that this report will contribute to the literature, since WBS is a rare disorder and the follow-up of the cognitive development of children with WBS has not been sufficiently addressed in the literature.

## Figures and Tables

**Figure:**
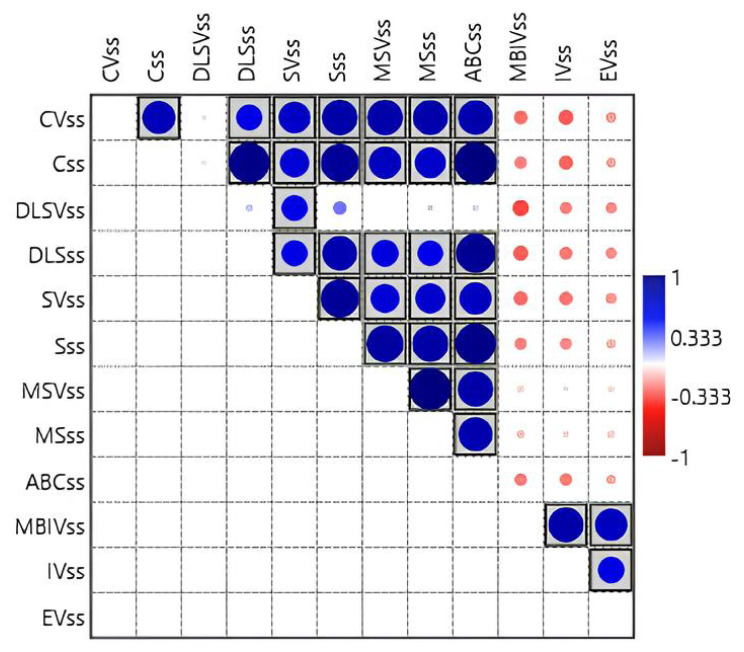
Correlation graph of the scale scores. Significant correlations are shown (p < 0.05). Red indicates a negative relationship and blue indicates positive. CVss, Communication V-scale score; Css, Communication Standard score; DLSVss, Daily Living Skills V-scale score; DLSss, Daily Living Skills Standard score; SVss, Socialization V-scale score; Sss, Socialization Standard score; MSVss, Motor Skills V-Scale score; MSss, Motor Skills Standard score; ABCss, Adaptive Behavior Composite Standard score; MBIVss, Maladaptive Behavior Index V-scale score; IVss,. Internalizing V-scale score; EVss, Externalizing V-scale score.

**Table 1:** Clinical features of the 12 children with WBS.

Variable		n (%)
Characteristics facial features		12 (100)
Ocular and visual problems	Hyperopia	2 (16.7)
	Strabismus	5 (41.7)
	Vision loss	1 (8.3)
Auditory loss		1 (8.3)
Chronic otitis media		3 (25.0)
Hypersensitivity to sound		11 (91.7)
Delay speech acquisition, followed by excessive talking		9 (75.0)
Failure to thrive height and weight <5th percentile		9 (75.0)
Visuospatial problems		9 (75.0)
Cardiovascular disease Peripheral PS (n = 10), supravalvular AS (n = 3), PFO (n = 3), VSD (n = 2), PDA (n = 1), mitral regurgitation (n = 1), secondary ASD (n = 1)		12 (100)
Hypertension		3 (25.0)
Genitourinary problems Urinary incontinence (n = 3), nephrocalcinosis (n = 2), overactive bladder (n = 1), undescended testis (n = 1), left renal agenesis (n = 1), subcoronal hypospadias (n = 1)		6 (50.0)
Hypercalcemia		6 (50.0)
Hypercalciuria		4 (33.3)
Endocrine problems	Hypothyroidism	7 (87.5)
	Short stature	1 (12.5)
Connective Tissue Abnormalities Hoarse voice (n = 7), inguinal hernia (n = 4); joint laxity (n = 4); joint limitation (n = 2), hypotonicity (n = 2)		12 (100)
Gastrointestinal problems Prolonged colic (n = 11), chronic constipation (n = 11), GER (n = 8), vomiting (n = 6), swallowing difficulties (n = 2), PEG (n = 1)		12 (100)
Feeding problems		7 (58.3)
Musculoskeletal problems Balance problems (n = 3), joint limitation (n = 2), kyphoscoliosis (n =1), scoliosis (n = 1), hand movement limitation (n = 1), trigonocephaly (n = 1), syndactyly (n = 1)		7 (58.3)
Dental anomalies		5 (41.7)
Neurological problems		1 (8.3)

PS: Pulmonary stenosis, AS: aortic stenosis, PFO: patent foramen ovale, VSD: ventricular septal defect, PDA: patent ductus arteriosus, ASD: atrial septal defect.

**Table 2:** Vineland-II scores in the 12 children with WBS.

Variable	Mean ± SD
Communication V-scale score	22.5 ± 7.3
Communication Standard score	62.7 ± 17
Daily Living Skills V-scale score	23.75 ± 6.0
Daily Living Skills Standard score	61.3 ± 14
Socialization V-scale score	25.7 ± 7.2
Socialization Standard score	65.7 ± 15.7
Motor Skills V-scale score	16.1 ± 3.8
Motor Skills standard score	59.2 ± 8
Adaptive Behavior Composite Standard score	60.4 ± 12.4
Maladaptive Behavior Index V-scale score	18.9 ± 1.4
Internalizing V-scale score	19.5 ± 1.4
Externalizing V-scale score	17.25 ± 1.7

**Table 3:** Maladaptive behaviors of the 12 children with WBS.

Variables	n (%)
Behavioral problems Short attention span (n = 5), enuresis nocturna-diurna (n = 4), picky eating (n = 3), stubborn (n = 3), tantrums (n = 2), nail biting (n = 2), headbanging (n = 1), thumb sucking (n = 1), bruxism (n = 1), aggression (n = 1)	8 (66.7)

Neurodevelopment problems Attention-deficit hyperactivity disorder (n = 3), Autism spectrum disorder (n = 1), Obsessive compulsive disorder (n = 1)	4 (33.3)
Anxiety	5 (41.7)
Feeding problems	8 (66.7)
Sleep disorders	4 (33.3)

**Table 4:** Comparison of the scores for patients with and without neurodevelopmental disease.

Variable	Neurodevelopmental disease		p-value
	Yes (n = 4)	No (n = 8)	
	Median (min–max)	Median (min–max)	
Communication V-scale score	24.5 (12–25)	24.5 (9–32)	0.668
Communication Standard score	60.5 (31–64)	64.5 (45–100)	0.269
Daily Living Skills V-scale score	21.5 (15–24)	26.5 (14–34)	0.148
Daily Living Skills Standard score	55 (36–62)	61 (55 – 94)	0.106
Socialization V-scale score	25 (15–27)	25.5 (16–40)	0.387
Socialization Standard score	63.5 (44–68)	68 (38–90)	0.394
Motor Skills V-scale score	15.5 (15–16.11)	17.05 (7–22)	0.490
Motor Skills Standard score	57.6 (56–59.22)	61.1 (40–72)	0.301
Adaptive Behavior Composite Standard score	58.5 (40–64)	62 (43–85)	0.396
Maladaptive Behavior Index V-scale score	20.5 (19–21)	18.9 (16–19)	0.007
Internalizing V-scale score	20.5 (20–22)	19.5 (17–20)	0.009
Externalizing V-scale score	19 (18–20)	17.1 (14–17.25)	0.006

Bold values denote statistical significance at p < 0.05.

**Table 5:** Comparison of the GMCD results and scores.

Variable	GMCD indicated delay		p-value
	Yes	No	
	Median (min–max)	Median (min–max)	
Communication Standard score	59 (31–74)	77 (65–100)	0.021
Daily Living Skills Standard score	57 (36–69)	75 (64–94)	0.021
Socialization Standard score	63 (38–81)	87 (72–90)	0.020
Motor Skills Standard score	57 (40–72)	64 (63–69)	0.049
Adaptive Behavior Composite Standard score	56 (40–70)	73 (63–85)	0.033
Maladaptive Behavior Index V-scale score	19 (17–21)	18.9 (16–18.87)	0.157
Internalizing V-scale score	20 (17–22)	19.5 (17–19.5)	0.184
Externalizing V-scale score	17.25 (14–20)	17.25 (17–17.25)	0.637

Bold values denote statistical significance at p < 0.05.

## Footnotes

1 Faul F, Erdfelder E, Buchner A, Lang AG. “G* Power Version 3.1. 7 [computer software],” Uiversität Kiel, Germany; 2013.

2 Vineland Uyum Davranış Ölçeği (Vineland-II) – Araştırma Formunun Doğumdan 8 yaşa Kadar Olan Türk Çocukları İçin Norm, Güvenirlik ve Geçerlik Çalışması (2008). Türkiye [online]. Website https://kitaplar.ankara.edu.tr/xmlui/bitstream/handle/20.500.12575/41440/%E2%80%9CVineland%20uyum%20davran%C4%B1%C5%9F%20%C3%B6l%C3%A7e%C4%9Fi.pdf?sequence=1&isAllowed=y [accessed March 2022].
